# Supplementary material for: A Vaccine Displaying a Trimeric Influenza-A HA Stem Protein on Capsid-Like Particles Elicits Potent and Long-Lasting Protection in Mice
Source: Vaccines (Basel). 2020 Jul 15;8(3):389. doi: 10.3390/vaccines8030389 (PMC7564254; doi:10.3390/vaccines8030389)
Supplement: Supplementary file 1 [file vaccines-08-00389-s001.pdf]

Supplementary Data

A vaccine displaying a trimeric influenza-A HA stem protein on capsid-like particles elicits potent and long-lasting protection in mice

Susan Thrane[1], Kara-Lee Aves[1], Ida E. M Uddbäck[1], Christoph M. Janitzek[1], Julianna Han[2], Yuhe R. Yang[2], Andrew B. Ward[2], Thor G. Theander[1,3], Morten A. Nielsen[1,3], Ali Salanti[1,3], Allan R. Thomsen[1], Jan P. Christensen[1], Adam F. Sander[1,3,*]

[1] Department of Immunology and Microbiology, University of Copenhagen, 2200 Copenhagen, Denmark.

[2] Department of Integrative Structural and Computational Biology, The Scripps Research Institute, La Jolla, CA 92037, USA

[3] AdaptVac Aps, Agern Alle 1, 2970 Hørsholm, Denmark.

[*] Correspondence: asander@sund.ku.dk

a

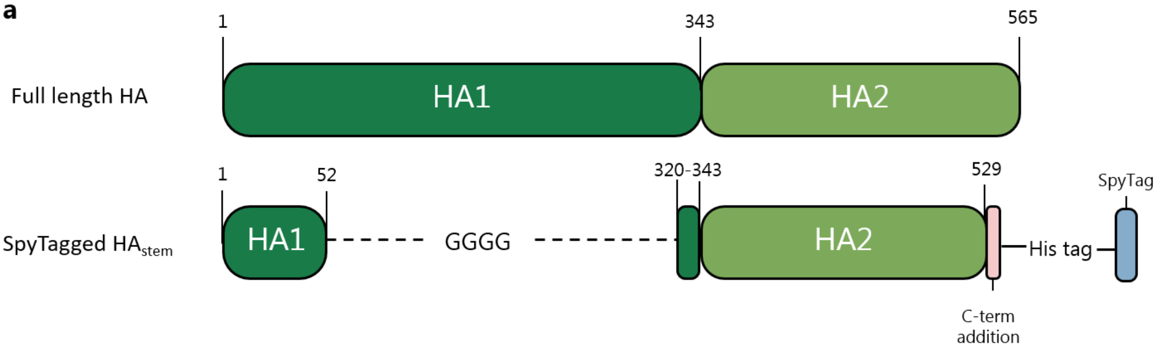

b

```
                                 1                    21                   41
SpyTagged HAstem                 MKVKLLVLLCTFTATYADTI CIGYHANNSTDTVDTVLEKN VTVTHSVNLLENGGGGKYVC
A/Brisbane/59/2007 (H1N1)        MKVKLLVLLCTFTATYADTI CIGYHANNSTDTVDTVLEKN VTVTHSVNLLENSHNGKYVR
A/Puerto Rico/8/1934 (H1N1)      MKANLLVLLCALAAADADTI CIGYHANNSTDTVDTVLEKN VTVTHSVNLLEDSHNGKYVR

                                 61                   81                   101
SpyTagged HAstem                 SAKLRMVTGLRNKPSKQSQG LFGAIAGFTEGGWTGMVDGW YGYHHQNEQGSGYAADQKST
A/Brisbane/59/2007 (H1N1)        SAKLRMVTGLRNIPSIQSQG LFGAIAGFIEGGWTGMVDGW YGYHHQNEQGSGYAADQKST
A/Puerto Rico/8/1934 (H1N1)      SAKLRMVTGLRNIPSIQSRG LFGAIAGFIEGGWTGMIDGW YGYHHQNEQGSGYAADQKST

                                 121                  141                  161
SpyTagged HAstem                 QNAINGITNKVNSVIEKMNT QYTAIGCEYNKSERCMKQIE DKIEEIESKIWCYNAELLVL
A/Brisbane/59/2007 (H1N1)        QNAINGITNKVNSVIEKMNT QFTAVGKEFNKLERRMENLN KKVDDGFIDIWTYNAELLVL
A/Puerto Rico/8/1934 (H1N1)      QNAINGITNKVNTVIEKMNI QFTAVGKEFNKLEKRMENLN KKVDDGFLDIWTYNAELLVL

                                 181                  201                  221
SpyTagged HAstem                 LENERTLDFHDSNVKNLYEK VKSQLKNNAKEIGNGCFEFY HKCNDECMESVKNGTYDYPK
A/Brisbane/59/2007 (H1N1)        LENERTLDFHDSNVKNLYEK VKSQLKNNAKEIGNGCFEFY HKCNDECMESVKNGTYDYPK
A/Puerto Rico/8/1934 (H1N1)      LENERTLDFHDSNVKNLYEK VKSQLKNNAKEIGNGCFEFY HKCDNECMESVRNGTYDYPK

                                 241                  261                  281
SpyTagged HAstem                 YSEESKLNREKIDGVKLESM GVYQIEGRHHHHHHGGAHIV MVDAYKPTK
A/Brisbane/59/2007 (H1N1)        YSEESKLNREKIDGVKLESM GVYQI--------------- ---------
A/Puerto Rico/8/1934 (H1N1)      YSEESKLNREKVDGVKLESM GIYQI--------------- ---------
```

Supplementary Fig. 1 Sequence comparison of SpyTagged HA$_{stem}$ with wild type HA proteins

a. Graphical depiction of Spytagged HA$_{stem}$ antigen compared to full length A/Brisbane/59/2007 HA.

b. Sequence alignment of Spytagged HA$_{stem}$ with corresponding amino acid segments from A/Brisbane/59/2007 and A/Puerto Rico/8/1934 H1N1 viral strains. Residues engineered into SpyTagged HA$_{stem,}$ which are not present in the A/Brisbane/59/2007 parental protein, are highlighted in green. Non-conserved amino acid residues between the A/Brisbane and A/PR8 strains are colored red. There is a 93% pairwise identity between the A/Brisbane and A/PR8 HA$_{stem}$ sequences compared to an 86.5% identity between the full-length HA proteins of the two strains.

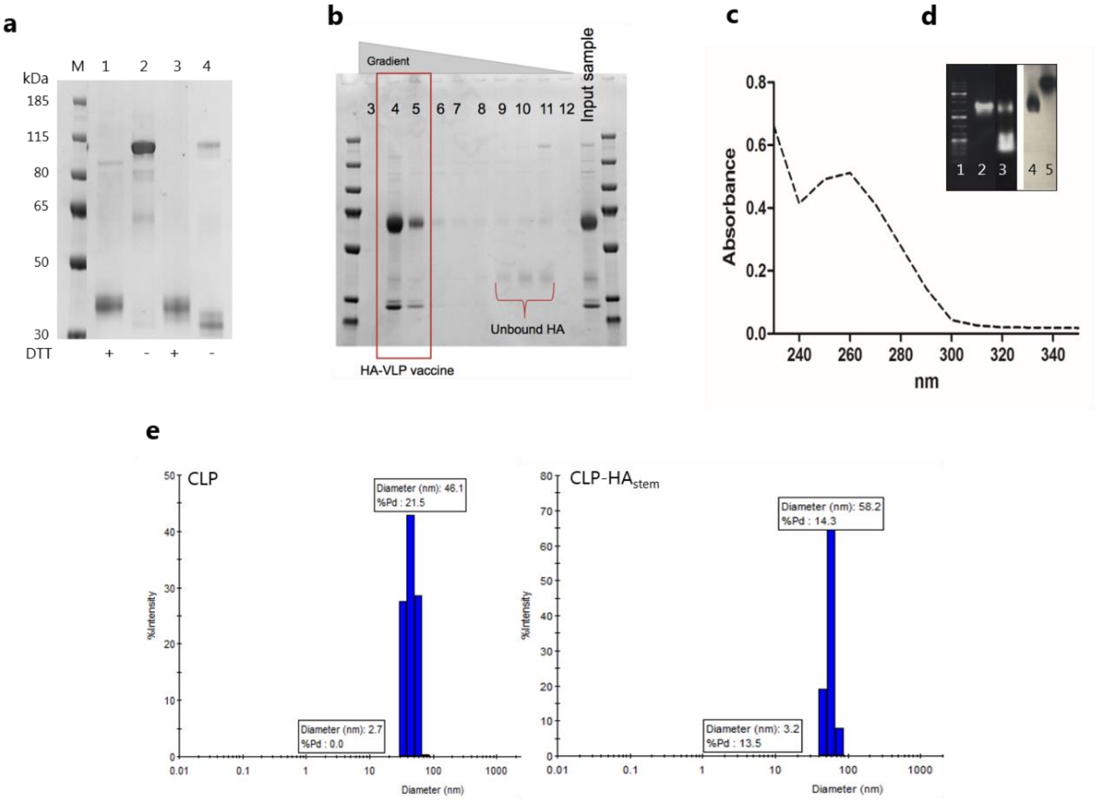

Supplementary Fig. 2 Characterization of purified HA$_{stem}$ and CLP-HA$_{stem}$.

a Purification of HA$_{stem}$ trimer via immobilized metal affinity chromatography followed by size exclusion chromatography. SDS-PAGE run under reducing (+DTT) and non-reducing (-DTT) conditions, containing a fraction eluted early during gel filtration, and thus containing predominantly trimeric HA$_{stem}$ (*lane 1 and 2),* and a fraction eluted later on, containing predominantly monomeric HA$_{stem}$ *(lane 3 and 4).* **b** Purification of CLP-HA$_{stem}$ by density gradient ultracentrifugation. Fraction 3 (high density) to 12 (low density) run on reducing SDS-PAGE alongside input sample taken before ultracentrifugation. Fraction 4 and 5 were pooled and used for the immunization studies. **c** UV spectrum of purified CLP. RNA absorption dominates (260nm). **d** Agarose gel analysis of AP205 CLP. 1kb DNA size marker (*lane 1*), AP205 CLP input (*lane 2 and 4*), Urea denatured AP205 CLP (*lane 3+5*). *Left* ethidium bromide stained gel, *right* coomassie stained gel. **e** Dynamic light scattering (DLS) analysis of uncoupled CLP *(left)* and CLP-HA$_{stem}$ vaccine *(right).*

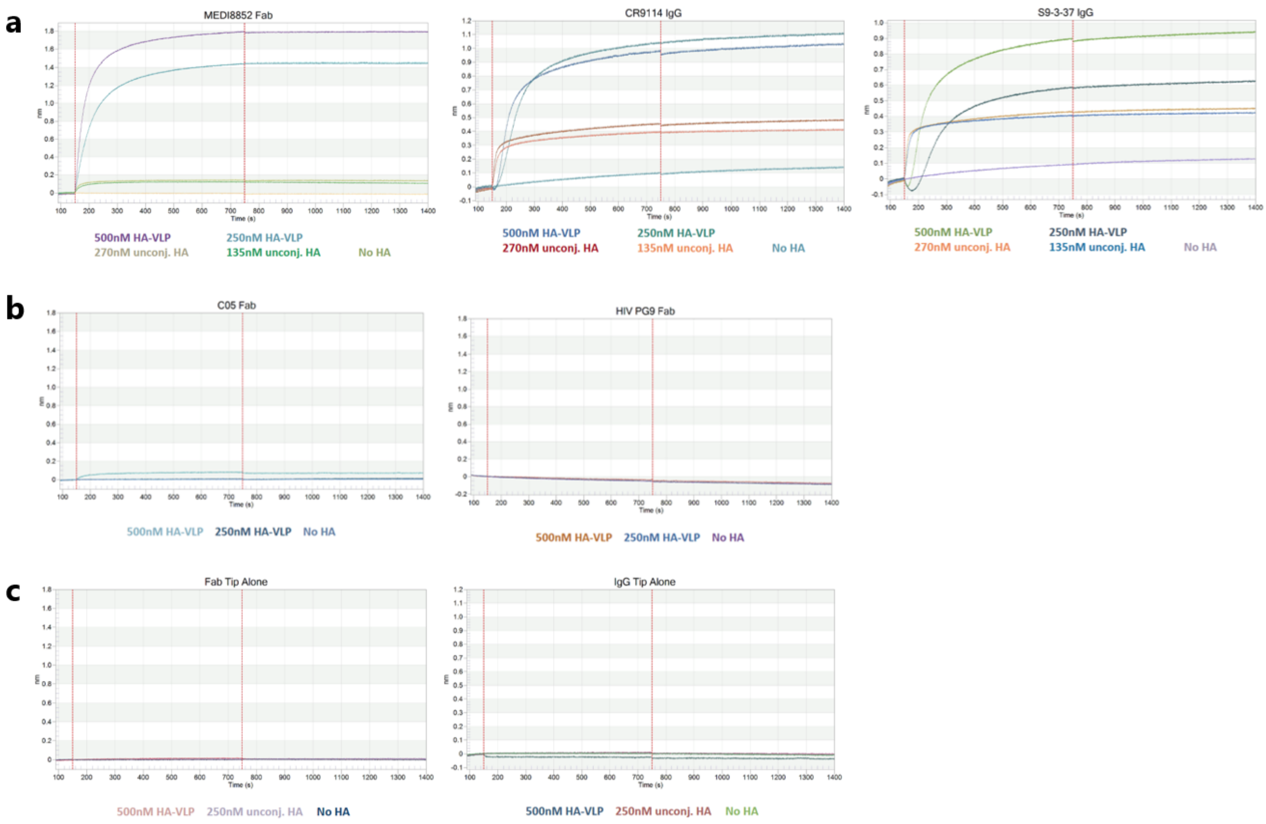

Supplementary Fig. 3 Binding of bnAb to HA_{stem} and CLP-HA_{stem}.

Bio-layer interferometry analysis of the binding of **a** MEDI8852 Fab *(left),* CR9114 IgG *(center)* and S9-3-37 IgG *(right)* bnAbs; **b** C05 Fab (HA-head specific) *(right)* and HG9 Fab (HIV specific) *(left)*; **c** Fab *(left)* or IgG *(right)* tip alone, to soluble HA_{stem} (270mM and 135nM) and CLP-HA_{stem} (500nM and 250nM). Each plot is one representative of 3 independently performed experiments.

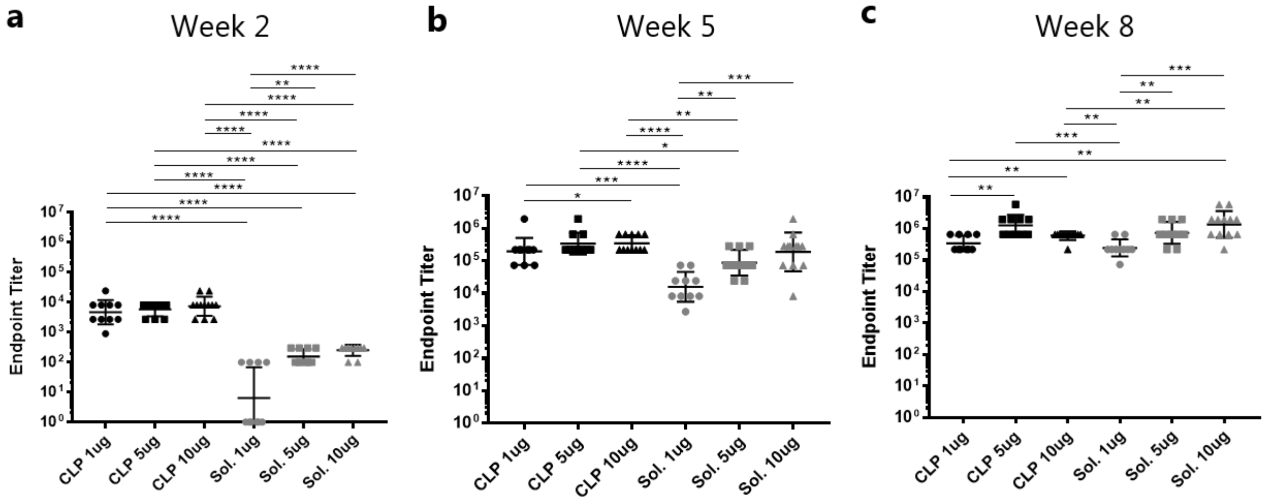

Supplementary Fig. 4 HA_{stem} specific IgG titres from dose escalation study

ELISA measurements of HA_{stem} specific IgG titres from serum taken at **a** Week 2, **b** Week 5 and **c** Week 8. Cut off was set to OD_{450nm} of 0.2. Each dot represents one animal. Horizontal lines indicate geometric mean of the group and vertical lines indicate the standard deviation. *p<0.05; **p<0.005; ***p<0.0005, ****p>0.00005.
